# Skin Microbiota in Atopic Dermatitis

**DOI:** 10.3390/ijms23073503

**Published:** 2022-03-23

**Authors:** Dora Hrestak, Mario Matijašić, Hana Čipčić Paljetak, Daniela Ledić Drvar, Suzana Ljubojević Hadžavdić, Mihaela Perić

**Affiliations:** 1 Department of Intercellular Communication, Center for Translational and Clinical Research, University of Zagreb School of Medicine, Šalata 2, 10000 Zagreb, Croatia; dora.hrestak@mef.hr (D.H.); hana.paljetak@mef.hr (H.Č.P.); mihaela.peric@mef.hr (M.P.); 2 Department of Dermatology and Venereology, University Hospital Centre Zagreb, University of Zagreb School of Medicine, Kišpatićeva 12, 10000 Zagreb, Croatia; daniela.ledic.drvar1@zg.t-com.hr (D.L.D.); suzana.ljubojevic@gmail.com (S.L.H.)

**Keywords:** atopic dermatitis, skin microbiota, dysbiosis

## Abstract

The skin microbiota represents an ecosystem composed of numerous microbial species interacting with each other, as well as with host epithelial and immune cells. The microbiota provides health benefits to the host by supporting essential functions of the skin and inhibiting colonization with pathogens. However, the disturbance of the microbial balance can result in dysbiosis and promote skin diseases, such as atopic dermatitis (AD). This review provides a current overview of the skin microbiota involvement in AD and its complex interplay with host immune response mechanisms, as well as novel therapeutic strategies for treating AD focused on restoring skin microbial homeostasis.

## 1. Human Skin Microbiota

All microorganisms residing in a multicellular host form its microbiota. Microbiota helps to maintain the balance (homeostasis) of the host system, contributes to immune responses and promotes tissue repair. Disruption of microbiota balance often results in inflammation or infection that can lead to various pathophysiological conditions and diseases. Additionally, imbalance leads to a microbial shift, often reducing beneficial species, and thereby causing dysbiosis. While the predominant part of the human microbiota is located in the gastrointestinal tract, remaining microorganisms are unequally distributed across the body, including the skin.

**Figure 1:**
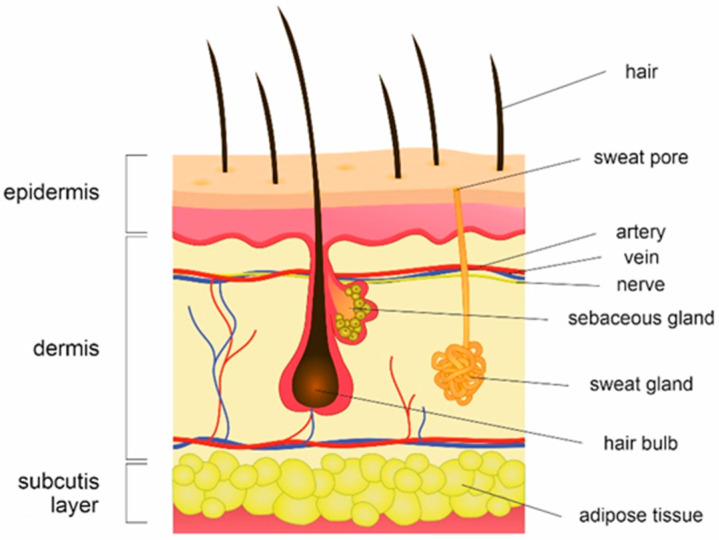
Schematic representation of human skin structure consisting of three main layers: epidermis, dermis and subcutis. The outermost skin layer, epidermis, is composed of terminally differentiated keratinocytes that enable continuous skin renewing, held together by corneodesmosomes and mortar lipids. Epidermis is supported by the collagen-bound dermis that provides a home for nerves, blood, lymph vessels, mast cells and other structures (i.e., sweat glands, hair follicles), and subcutis consisting of adipose tissue. Skin regions vary in terms of topography, temperature, salt content and acidity (pH) and are, based on their features, categorized into three major groups: moist sebaceous and dry.

The skin is the largest organ of the human body, forming a protective barrier that prevents infections from environmental pathogens, and regulates body temperature, prevents water loss and triggers pain response. The human skin (Figure 1) can also be perceived as an ecosystem, where different parts of the body represent different habitats for bacteria, fungi, viruses and archaea. Microorganisms colonize the skin surface according to the spectrum of preferences, forming units that contribute to the body’s immune system and protect against other pathogenic life forms. Each microbe has adapted to the physicochemical characteristics of its habitat—a behavior similar to that of Earth’s flora and fauna. In addition, because skin has little nutritive value aside from lipids and proteins, the main requirement for microbe survival is their utilization of resources found in the *stratum corneum* and/or sebum, such as amino acids or urea [1].

Bacterial phyla which can be found on the healthy human skin are *Actinobacteria*, *Firmicutes*, *Proteobacteria* and *Bacteriodetes* [2], with an emphasis on *Staphylococcus*, *Corynebacterium* and *Propionibacterium* genera that comprise more than 60% of the bacterial skin population [3]. The composition of skin microbiota is highly dependent on the characteristic physiology of the skin site, and specific bacterial taxa were found to inhabit dry, moist and sebaceous microenvironments [4]. Dry areas (forearm, buttock, various parts of the hand) are the most diverse skin sites, reported to contain greater bacterial diversity than the gut or the oral cavity of the individual [5], and harboring numerous phylotypes including β-*Proteobacteria*, *Corynebacteria* and *Flavobacteriales* [2]. Moist skin sites (nostril, armpit, navel, toe webs, inner elbow, groin, cubital and popliteal fossa, palms and soles) provide thermally stable, warm environments, with *Corynebacteria* and *Staphylococci* species being the most abundant organisms that can withstand humid conditions [4]. Finally, sebaceous areas (forehead, retroauricular area, lateral sides of the nostrils, back) show the lowest bacterial diversity and are populated mostly by lipophilic *Propionibacteria* and *Staphylococci* species [3,4,6]. Although the skin microbiota encounters frequent perturbations due to the constant environmental changes, longitudinal sampling revealed its stability over a 2-year period, with the microbial communities at sebaceous sites being most persistent and those at foot sites the least stable [7,8]. As opposed to bacterial communities, the composition of fungal communities was reported similar across the body sites despite different skin physiology [9]. Studies on fungal communities distinguish the predominance of the genus *Malassezia* on healthy human skin, specifically on the chest and arms (*M. globosa*), the trunk (*M. sympodialis*) and on facial sites (*M. restricta*) [9,10,11]. Foot sites were colonized by diverse communities of *Malassezia*, *Aspergillus*, *Cryptococcus*, *Rhodotorula*, *Epicoccum* and others, which display lower stability over time [9,12]. Several studies show that normal skin microbiota also included *Candida* [12,13]. However, organisms belonging to the kingdom of fungi represent the least abundant inhabitants of the skin [8]. In contrast to bacterial and fungal communities, no healthy human skin core virome was found conserved among individuals [8]. Foulongne et al. reported a metagenomic study that displayed multiple eukaryotic virus families, namely, *Polyomaviridae*, *Papillomaviridae* and *Circoviridae* [14]. A more recent study by Hannigan et al. demonstrated the presence of *Adenoviridae*, *Anelloviridae*, *Circoviridae*, *Herpesviridae*, *Papillomaviridae* and *Polyomaviridae* [15]. However, a core phageome consisting of *Propionibacterium*, *Staphylococcus*, and *Streptococcus* bacteriophages was recently identified. These phages are obligate partners to the most abundant bacterial species and are therefore site-specific [8].

The stability of skin microbial communities varies between age groups, the diversity being inversely proportional to maturity [16,17]. Initial colonization of the skin in newborn babies depends greatly on the delivery mode and mother’s bacteria to which the neonates are exposed during labor [18]. The skin microbiota of children under 12 years comprises mostly *Streptococcus*, *Granulicatella*, *Gemella*, *Rothia* and *Haemophilus* bacterial genera [16], together with highly diverse fungal communities of *Ascomycota* (*Aspergillus*, *Epicoccum*, *Phoma*), *Cladosporium* and *Cryptococcus*, and a low abundance of *Malassezia* [17]. During puberty, the structure of the skin changes, as the increased hormone levels stimulate additional production of sebum, thus favoring the expansion of lipophilic microbiota. The *Streptococcus* genus is gradually replaced with *Propionibacterium* and *Corynebacterium*, with *Malassezia* as a predominate fungal constituent of adult skin microbiota [16,17].

## 2. Atopic Dermatitis

Atopic dermatitis (AD) is one of the most common, chronic, inflammatory skin diseases of the modern world. Additionally known as ‘eczema’, this chronic recurrent disorder is characterized by an intense itching sensation and eczematous lesions. Acute lesions are manifested as bright erythema, oedema and oozing, while chronic lesions present as xerosis, lichenification and residual dyspigmentation. In comparison to healthy controls, both non-lesional and lesional AD skin show higher transepidermal water loss (TEWL) and pH values [19] that corelate with disease severity and predisposition [20,21]. Distributional and morphological characteristics generally relate to age, whilst some phenotypes show predisposition for certain body sites. Although most frequently manifested throughout the first year of life, the disease can occur at any age. Early-onset AD is sub-classified based on age groups as infantile (<2 years), childhood (2–12 years) or adolescent (12–18 years). Additionally, children with AD are more prone to developing other atopic diseases than children without AD [22,23], and AD can be a first step in the sequential development of other atopic manifestations later in life (food allergy, asthma and allergic rhinitis), in the process of the “atopic march” [24,25,26]. Adult-onset occurs after 18 years of age. Recent studies indicate a third class of AD that occurs after the age of 60 and is, therefore, termed the elderly-type AD, sub-labelled as elderly-onset, relapsing and continuous [27,28,29].

Diagnostic criteria established for AD consist of basic features, such as pruritus, lichenification, and chronically relapsing course, and personal and/or family atopic history as well as minor features take into consideration less specific symptoms [30,31,32]. Disease severity can be measured by various tools, estimating the extent of affected areas and multiple types of symptoms to help assess and score clinical signs. Typically preferred are the Scoring of Atopic Dermatitis Index (SCORAD) and the Eczema Area and Severity Index (EASI) [33,34,35].

## 3. Pathogenesis of Atopic Dermatitis

There are numerous risk factors associated with the development of AD, from genetic predisposition to environmental conditions. Family history of atopic diseases presents the most prominent risk factor for AD. Research shows that the occurrence of any atopic disease in one of the parents increases 1.5-fold the possibility a child developing AD. Moreover, the risk is further increased 3-fold and 5-fold, respectively, if one or both parents suffer from AD [36]. Several environmental risk factors have been proposed to enhance the prevalence of AD, such as living in an urban setting, low UV light exposure, dry climate, Western diet, repeated exposure to antibiotics in early childhood, as well as high level of household education [37].

Pathophysiology of AD is very complex and not yet completely elucidated. Multiple contributing factors, including epidermal barrier impairment, immune dysregulation and alteration of skin microbiota, contribute to the disease. The integration of these factors, with their different intensities and combinations, is thought to cause the varying clinical presentations of AD [38].

### 3.1. Epithelial Barrier Dysfunction and Immune Dysregulation

The skin barrier plays a major role in the protection from commensal and pathogen penetration, forming a thin line between health and disease. Several factors contribute to epithelial barrier dysfunction in AD, including mutations of genes that encode structural and functional proteins of the epidermis and epigenetic modifications affecting the regulation of immune response and inflammatory processes [39]. Filaggrin is one of the key epidermal proteins for production of a natural moisturizing factor and is essential for maintaining *stratum corneum* hydration. Mutations in the filaggrin gene are found in about half of patients with moderate to severe disease [39] and can be associated with early-onset AD [40]. Deficit in filaggrin production also results in aberrant keratinocyte differentiation and insufficient skin lipid content [41]. Skin lipids (e.g., ceramides, free fatty acids and cholesterol) are vital for the maintenance of the epidermal barrier function, and thus are responsible for prevention of TEWL and penetration of irritants, allergens and microbes. Disbalance in the composition of skin lipids, as well as the reduction of the lipid content, are associated with the development of AD [19,42].

**Figure 2:**
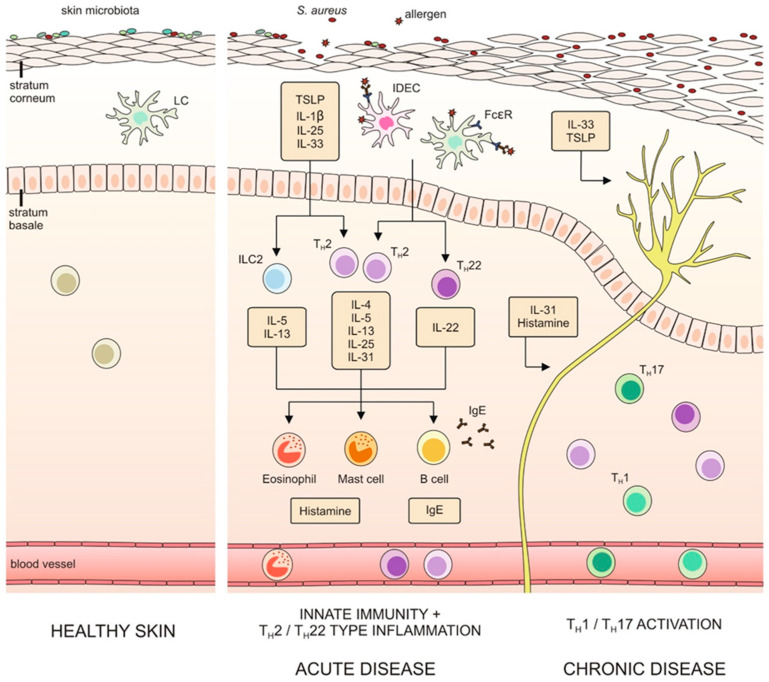
Epithelial barrier dysfunction, immune dysregulation and skin microbiota dysbiosis in initiation and progression of AD. Epithelial barrier dysfunction and stress from environmental and mechanical factors lead to skin barrier damage and enhanced epidermal permeability, which in turn increases microbial and allergen contact with the cutaneous immune system. Damaged keratinocytes activate immune mechanisms by releasing proinflammatory cytokines (IL-1β, TSLP, IL-25, IL-33) and chemokines, mobilizing innate lymphocyte subsets and skin-resident dendritic cells (DCs). DCs attract and prime naive T-cells, promoting T_H_2/T_H_22 cell responses and inducing inflammation process. Type 2 cytokines (IL-4, IL-5, IL-13, IL-25) drive the inflammation, recruiting and activating other types of immune cells, such as eosinophils, mast cells and B-cells. The secreted molecules and proinflammatory cytokines act directly on cutaneous nerves and contribute to pruritus. Moreover, the inflammation further disrupts skin barrier and favors colonization by pathogens (*S. aureus*), additionally inducing keratinocyte damage and boosting T_H_2-type response, thus supporting the disease cycle. The activation of T_H_1/T_H_17 cell responses in chronic disease induce tissue remodeling, increasing skin thickness and lichenification. IDEC—inflammatory dendritic epidermal cell, ILC2—group 2 innate lymphoid cell, LC—Langerhans cell, T_H_1—T_H_1 cell, T_H_2—T_H_2 cell, T_H_17—T_H_17 cell, T_H_22—T_H_22 cell.

Epidermal barrier disruption stimulates keratinocytes to release chemokines (thymus and activation-regulated chemokine (TARC), macrophage-derived chemokines (MDC)) and cytokines (IL-1β, IL-25, IL-33 and thymic stromal lymphopoietin (TSLP)). This leads to infiltration of leukocytes, primarily dendritic cells (DC), eosinophils and T-cells [43], initiation of T_H_2 cell responses, as well as activation of skin-resident group 2 innate lymphoid cells (ILC2s), thus inducing inflammation [44,45,46]. T_H_2 cells release IL-4, IL-5, IL-13, IL-25 and IL-31, which in turn activate B-cells to produce IgE molecules. ILC2s also release IL-5 and IL-13 and can provide an additional boost to type 2 immunity and IgE production [46]. Together with T_H_2 cells, IL-22-secreting T_H_22 cells play an important part in the initiation and acute phase of AD [45], while the switch to T_H_1/T_H_17 response characterizes a chronic disease [47]. Keratinocyte-expressed chemokines also recruit different dendritic cell subtypes, including inflammatory dendritic epidermal cells (IDECs) and Langerhans cells, which express high-affinity immunoglobulin-ε receptors (FcεR1s). Binding of IgE antibodies to these receptors facilitates the uptake of allergens and triggers hypersensitivity reactions [48]. Additionally, epithelial cell-derived IL-33 and TSLP and type 2 cytokines can directly activate itch-sensory neurons to induce pruritus [49,50]. The dynamic interplay between epithelial barrier dysfunction, type 2 immunity and pruritus is schematically shown on Figure 2.

### 3.2. Dysbiosis of Skin Microbiota

The skin of patients with AD reveals considerable anomalies of the microbial communities when compared to the microbiota of healthy subjects; however, it is not certain whether these changes are the cause or the result of epidermal barrier dysfunction and immune dysregulation. Shi et al. showed that 20 genera usually found on healthy individuals also occupied the skin of AD patients [16]. However, the skin in AD is characterized by an expressed microbial imbalance and reduced diversity, specifically manifested as a decrease in the genera *Cutibacterium*, *Streptococcus*, *Acinetobacter*, *Corynebacterium* and *Prevotella*, and a marked increase of *Staphylococcus*, especially of *S. aureus* [51]. The reduced microbial diversity is particularly evident during severe flares of the disease, with the reported composition of *Staphylococcus* usually reduced to a single *S. aureus* strain [18]. The treatment and recovery reverted the microbiota to its pre-flare configuration [51].

**Figure 3:**
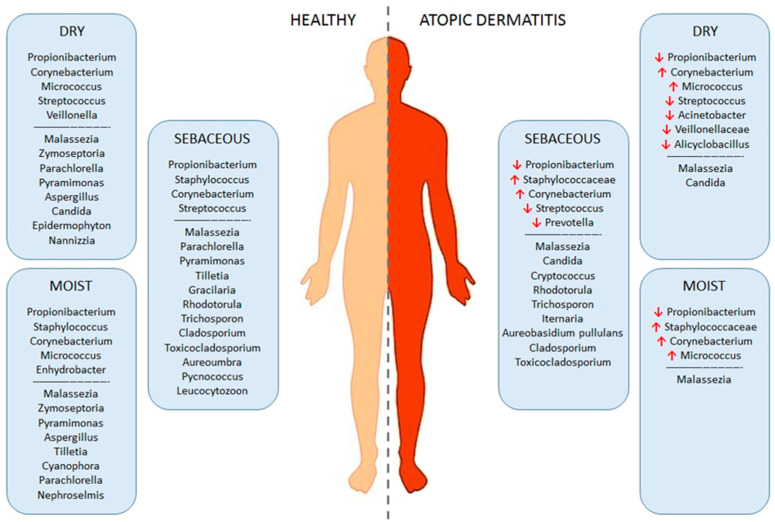
Comparison of microbiota composition between healthy skin and skin affected by AD.

The disequilibrium of skin commensals might not sound like an ominous occurrence, however, it instigates another set of problems. Because a balance normally ensures that commensals remain benign and/or beneficial, a microbial shift provides growth space for species whose greater numbers cause more harm than good (Figure 3). Consequently, newly dominant strains can trigger skin inflammation and diseases. *S. aureus* is the principal bacterial species associated with AD [52]. As an opportunistic pathogen, it is well adapted to adhere to the skin, disrupt the epithelial barrier and trigger the host immune system, in turn inducing skin inflammation [53,54]. While the carriage of *S. aureus* in healthy subjects is about 20%, the prevalence on the skin of patients with AD is increased and varies from 30% to 100%, depending on patient age, AD severity, as well as sampling and analysis methods [55]. Additionally, the reported abundance of *S. aureus* in patients with AD was 70% on lesional skin and 39% on non-lesional skin or healthy skin of the same patient, confirming the correlation between *S. aureus* and disease severity [55,56]. A recent study identified differences in *S. aureus* strain structure isolated from AD skin lesions from that of non-lesional skin: patients with severe AD tend to carry the clonal complex 1 (CC1) strains, whereas asymptomatic individuals carry the CC30 strains [57]. The chronic persistence of *S. aureus* on eczematous skin lesions and difficulties of eradicating it using antibiotics were found to be associated with the prevalence of staphylococcal biofilm communities on the skin of patients with AD. Indeed, a study by Di Domenico et al. confirmed the severity of AD can be linked to biofilm formation by *S. aureus* [56]. A number of studies focused on the interplay between *S. aureus*, epithelial barrier disruption and the immune system. Unlike healthy skin, skin in AD is more permissive to *S. aureus* colonization due to its reduced anti-microbial peptide (AMP) levels [58]. The reduced expression of AMPs, particularly defensins and cathelicidins, can be the result of T_H_2-derived IL-4 and IL-13 cytokines [59]. It was also shown that *S. aureus* adheres more strongly to AD skin, due to the filaggrin deficiency and deformed corneocytes of the *stratum corneum* [60]. Additionally, *S. aureus* produces a number of different proteolytic enzymes and toxins, as well as stimulates the expression of endogenous keratinocyte proteases, which can disrupt the integrity of the skin barrier and enable penetration through stratum corneum [61,62]. Other toxins from *S. aureus* can directly induce type 2 immune response by activating immune cells and triggering the expression of the inflammatory mediators such as IL-4, IL-13, IL-22 and TSLP [61].

Several reports have demonstrated that *Staphylococcus epidermidis* overgrowth can also be linked to AD pathogenesis. Usually perceived as a skin commensal essential in coordinating the maturation of the immune system and combating pathogens, *S. epidermidis* is, in certain conditions, able to contribute to the inflammatory reaction in AD [63]. Byrd et al. found that, unlike single strain *S. aureus* communities linked to severe AD flares, more heterogenous *S. epidermidis* strains dominated in patients with less severe symptom manifestations [18]. A number of studies reported that not only *Staphylococcus* strains incite development of AD. Previously known under the name *Propionibacterium acnes*, *Cutibacterium acnes* is one of the most widespread skin commensals, playing a role in the skin defense mechanisms [64]. However, *C. acnes* can also cause damage to the skin by enhancing *S. aureus* cytolytic activity, thus inducing proinflammatory cytokine production [65]. The porphyrin molecule coproporphyrin III (CIII) produced by *C. acnes* was found to induce *S. aureus* aggregation and biofilm formation, suggesting the cooperation between *C. acnes* and *S. aureus* [66].

In contrast to numerous studies on skin bacteria, reports on mycobiota diversity in AD are scarce. As in healthy subjects, *Malassezia* species (especially *M. globosa* and *M*. *restricta*) were predominant in patients with AD [12]. *M. restricta* dominated over *M. globosa* in patients with mild or moderate disease, while the ratio of the two species was equal in patients with severe disease [12,67]. *Malassezia* species were shown to penetrate the epithelial barrier of AD patients, causing activation of immune cells and skin inflammation [68]. In addition, *Malassezia* allergens can trigger a specific IgE response, contributing to the disease [68]. As for non-*Malassezia* fungi, *Candida albicans*, *Cryptococcus diffluens* and *Cryptococcus liquefaciens* were detected more often in patients with AD than in healthy subjects [12]. However, the role of these species in AD pathophysiology needs to be further elucidated.

**Figure 4:**
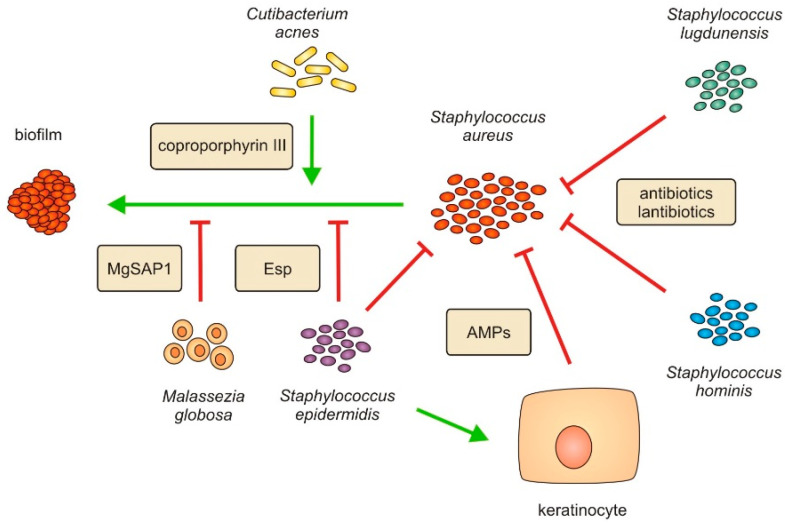
Interplay between *Staphylococcus aureus* and skin microbiota. Several members of *Staphylococcus* genus inhibit *S. aureus* growth and biofilm formation. *Staphylococcus lugdunensis* and *S. hominis* suppress colonization of *S. aureus* by secreting antibiotics and lantibiotics. *S. epidermidis* induces keratinocytes to produce anti-microbial peptides (AMPs) to eradicate *S. aureus*, as well as produces protease glutamyl endopeptidase (Esp) which inhibits formation of *S. aureus* biofilm. The MgSAP1 protease secreted by *Malassezia globosa* was shown to have a similar effect of disrupting *S. aureus* biofilm. In contrast, *Cutibacterium acnes* and its corpoporphyrin III molecule promote *S. aureus* activity and aggregation, thus inducing *S. aureus* biofilm formation.

Contrary to microbial communities being associated with inflammation incitement, there is evidence that the presence of some microbes negatively corelates with AD progression (Figure 4). Scharschmidt et al. confirmed that early exposure to commensal *Staphylococci* plays a role in antigen-specific tolerance that may prevent AD development in mice [69]. Those findings were corroborated by a study in which decreased susceptibility to AD was associated with early exposure of infants to *Staphylococci* commensals [70]. Studies reported several species of *Staphylococcus* genus suppressing *S. aureus* and its effect on disease progression. The coagulase-negative (CoNS) *S. epidermidis*, *Staphylococcus hominis* and *Staphylococcus lugdunensis* successfully inhibited *S. aureus* colonization and biofilm formation [71,72]. A similar effect was observed in more recent reports by Zipperer et al. and Nakatsuji et al., who showed that both *S. lugdunensis* and *S. hominis* produce lantibiotics that inhibit *S. aureus* growth [73,74]. Furthermore, an *in vitro* study demonstrated that a co-infection of *S. aureus* with the *Corynebacterium striatum*, in comparison to exclusive *S. aureus* infections, resulted in an *S. aureus* shift towards a commensal state [75]. In addition to the *C. striatum*, byproducts from glycerol fermentation by *Cutibacterium acnes* also showed *S. aureus* inhibition, without disrupting the skin microbiome balance [76]. Aside from metabolites of bacterial commensals, the MgSAP1 protease secreted by *Malassezia globosa* was shown to hydrolyze the *S. aureus* Protein A, thereby hindering its biofilm formation [77].

These findings provide evidence on the key role of skin microbiota in the pathogenesis of AD. However, the current scientific knowledge is still lacking, and future research efforts need to be directed towards fully understanding the composition of the microbial ecosystem of the human skin, as well as the complex interactions regulating the host–microbiota relationship in health and disease.

## 4. Treatment of Atopic Dermatitis

The AD management approach is based on disease severity, age and location. A continuous daily emollient application to relieve symptoms and enhance skin hydration represents a baseline therapeutical approach for both children and adults [78]. Preparations such as petrolatum, physiologic lipids or ceramide-based lipids are known to reduce TEWL and decrease bacterial colonization, which improves overall skin barrier function [79]. While standard aqueous creams show positive results in terms of symptom improvement, pH-modified moisturizers significantly alleviate symptoms in AD and serve as a useful treatment adjunct [80]. Emollients can also affect *Staphylococcus* species abundance and microbiota diversity [81]. However, in the acute phase of the disease, application of potent anti-inflammatory agents is required, with topical corticosteroids (TCS) representing the first-line anti-inflammatory treatment [78]. Although highly effective in improving AD symptoms, long-term use of corticosteroids is discouraged because of their side-effect profile and subsequent patient-compliance issues [78,82]. At the beginning of the 21st century, non-steroid topical calcineurin inhibitors (TCI) were introduced as an alternative to TCS treatments for AD. Macrolide derivatives tacrolimus and pimecrolimus are calcineurin inhibitors that prevent T-cell signal transduction and IL-2 transcription, thus suppressing inflammation [83,84]. Unlike TCS, TCIs are suitable for long-term treatment, and use of tacrolimus is recommended for the maintenance and reduction of relapses, often after initial corticosteroid treatment [78]. In addition, tacrolimus showed a positive impact on the skin microbiome in AD patients [85]. Antibiotics are included in AD treatment in cases of bacterial superinfection, but due to antibiotic resistance and the potential negative effect of antibiotics on commensal bacteria, this treatment method is not a long-term option [86]. Other therapeutic approaches include phototherapy with ultraviolet (UV) light, which can reduce AD recurrence. Narrow band ultraviolet B (nBUVB) phototherapy has been shown to decrease the *S. aureus* ratio in the skin microbiota [87]. Severe AD may require hospitalization and systemic immunosuppressive treatment with cyclosporine A, a short course of oral glucocorticoids, methotrexate, azathioprine and mycophenolic acid or, as an alternative, biologic therapy [88].

## 5. Probiotics and Prebiotics in Treatment of Atopic Dermatitis

Probiotics are live microorganisms with immunomodulatory features which provide beneficial effects on the host’s well-being. Although most probiotic applications have been targeted at gastrointestinal tract disorders, recent reports recognized a vast potential of utilizing probiotics for promoting skin health and managing various skin conditions. Over the last few years there have been scientific breakthroughs with reference to treating AD using oral or topical probiotic cultures. A recent animal study found that orally administered *Lactobacillus paracasei* KBL382 successfully ameliorates AD symptoms in mice via modulating the immune response and gut microbiota [89]. Similar research was conducted by Kwon et al., in which *Lactobacillus sakei* WIKIM30 isolated from kimchi and orally delivered to mice resulted in stimulation of Treg cell generation and suppression of T_H_2 inflammatory response, as well as in restoring the balance of gut microbiota [90]. Another animal study reported that administration of *Lactobacillus rhamnosus* IDCC 3201 tyndallizate (RHT3201) to mice resulted in less severe AD symptoms in comparison to controls, together with dose-dependent reductions in dermatitis scores [91]. Furthermore, experiments on mouse animal models by Kim et al. suggested oral administration of β-glucan and *Lactobacillus plantarum* LM1004 inhibited T_H_2 cell responses and activated Treg immunoregulatory functions, as well as increased relative abundance of butyrate-generating microorganisms in the gut [92].

Along with the research on animal models, clinical trials in humans also showed promising results regarding the oral use of probiotics in treating AD. One of the earliest publications in the field describes oral administration of a mixture containing two *Lactobacillus* strains (lyophilized *Lactobacillus rhamnosus* 19070-2 and *Lactobacillus reuteri* DSM 122460) to children with AD, throughout a period of six weeks in a double-blind placebo-controlled crossover study. The treatment provided a moderate improvement in the clinical severity of eczema [93]. *L. rhamnosus* [94,95,96] and *L. plantarum* CJLP133 [97] also displayed a positive treatment efficacy on AD during clinical trials on children. Another study reported successful treatment of AD patients using *Lactobacillus fermentum* VRI-033 PCC during a double-blind randomized placebo-controlled trial, with reduced SCORAD index and change in AD severity compared to placebo-treated individuals [98]. Moreover, Niccoli et al. efficiently treated pediatric AD patients with a lyophilized form of *Lactobacillus salivarious* LS01, reporting a significant decrease in SCORAD value and significant improvement in itching intensity when compared to the placebo control group, and both therapy benefits persisting after suspension of treatment [99]. Although typically tested oral probiotic formulations most often consisted of *Lactobacillus* strains, several studies investigated the positive effects of other bacterial strains or mixtures of different probiotic bacterial strains in management of AD. Matsumoto et al. reported that the administration of *Bifidobacterium animalis* subsp. *lactis* LKM512 alleviated itch in AD patients and considerably improved the quality-of-life scores when compared with the controls, suggesting an antipruritic effect of *B. animalis* [100]. A probiotic mixture of *Bifidobacterium lactis* CECT 8145, *B. longum* CECT 7347, and *Lactobacillus casei* CECT 9104 was reported to reduce the SCORAD index in AD patients compared with the control group [101], while a case report using a mixture of *Bifidobacterium lactis* HN019, *Lactobacillus acidophilus* NCFM, *Lactobacillus rhamnosus* HN001 and *Lactobacillus paracasei* LPC-37 described an evident response in treating severe AD with significant change in AD severity scores [102]. In fact, a meta-analysis suggested the administration of probiotics has a positive influence on the treatment of AD, with the greatest effect observed in studies using a mixture of different bacterial species [103].

Along with studies on oral probiotics, which indirectly influence skin diseases, a number of topical probiotic formulations have been proposed to ameliorate skin conditions by suppressing inflammation and restoring skin microbiota balance. Nakatsuji et al. demonstrated that the topical application of commensal skin bacteria is effective in protecting against pathogen species, with reduced *S. aureus* colonization due to selective anti-microbial peptides secreted by commensal coagulase-negative *Staphylococci*, improvement of clinical symptoms and decreased local inflammation [74,104]. A group of authors also conducted a first-in-human topical microbiome transplantation after collecting the commensal *Roseomonas mucosa* from healthy subjects. After a six-week therapy, significant decrease in SCORAD and pruritus was noted, as well as reduction in disease severity and no adverse effects or complications, which, consequently, signifies a lesser need for topical steroids [105]. Interestingly, several studies also explored the effects of the topical application of gut commensals for treating AD. A cosmetic lotion containing heat-treated *Lactobacillus johnsonii* was linked to the reduction of *S. aureus* load on the skin of adult AD patients and clinical improvement of AD symptoms [106]. Another report describes significant improvement in skin barrier integrity, erythema, scaling and pruritus in patients with AD after a 2-week topical administration of a cream consisting of sonicated *Streptococcus thermophilus* [107]. Finally, Gueniche et al. found that topical ointment containing lysate of *Vitreoscilla filiformis*, a Gram-negative bacterium found in thermal springs, which has traditionally been used in treating dermatological diseases, also resulted in clinical improvement in patients with AD [108]. Even though not technically probiotics, the probiotic bacteria preparations evidently have the ability to interact with the skin components and alleviate AD symptoms.

Unlike the studies on probiotics, the research on prebiotics and synbiotics (combination of probiotics and prebiotics) in AD treatment is relatively scarce. Chang et al. published a meta-analysis of six randomized controlled trial studies providing evidence to support the use of synbiotics composed of mixed strains of bacteria for the treatment of AD for children aged 1 year or older [109]. A double-blind randomized study by Passeron et al. performed on children with AD using a prebiotic preparation as well as synbiotic preparation (*Lactobacillus rhamnosus* Lcr35 plus prebiotic) showed both treatments significantly improved AD symptoms, with no significant difference noted between the two treatments [110]. A similar double-blind study was conducted by Aldaghi et al. on AD infants that were administered either a synbiotic mix containing *L. rhamnosus*, *L. euteri* and *B. infantis* or vitamin D3. The report confirmed both treatments significantly decreased SCORAD scores when compared to the control group [111].

Although further investigation is needed, the results shown in these studies (Table 1) warrant a potential interest in using probiotics and prebiotics as therapeutic treatment in the management of AD. However, it should be noted that these potential therapies are considered as Microbiotic Medicinal Products (MMPs). An MMP is any medicinal product containing living, dead, fragments or components of the microbiota (i.e., bacteria, yeasts, phages, etc.) with the purpose to prevent or treat human diseases through a pharmacological, microbiological, neurological, immunological or metabolic mode of action, or to make a medical diagnosis [112]. Although showing great promise for treating human disease, including AD, the development of these products (especially live biotherapeutic products, LBPs) is faced with many scientific, clinical and regulatory challenges [113]. The current set of requirements for a particular type of the product is not specifically or uniformly defined for LBPs at the global level, so the acceptance criteria for the product quality, efficacy, and safety are often unclear or inappropriate and eventually need to be adjusted to an individual product. The most obvious issue arises when considering the basic requirement for the pharmaceutical product, its sterility, that these therapies evidently cannot achieve. Additionally, the manufacturing of LBPs is complex due to batch-to-batch variations and many factors related to culture conditions and product stability (viability, shelf life, genetic stability) that could influence bacterial properties, consequently changing product efficacy or safety. Determining LBPs’ safety is different from other medicines since the product itself does not reach the systemic circulation, while its activities or metabolites may act directly or indirectly on (systemic) physiological functions of the host, so toxicity is not always directly related to the dosage. Furthermore, the translation of data from animals to humans is almost impossible due to the holobiont concept [114,115], a result of the coevolution of the microbiome and its human host that cannot be reproduced in animal species. Clinical efficacy for LBPs can only be proven in an independent trial of acceptable quality, using well-defined treatment conditions and dosage and with preliminary defined, validated endpoints. As currently no standard clinical trial format exists (products, target populations and application modes differ on a case-by-case basis), proving efficacy in a standardized manner is quite difficult to achieve. Many environmental factors (e.g., transport and storage conditions) as well as host-related factors (e.g., health status, stomach pH, interference with diet, the composition of the recipient microbiota, ethnicity, etc.) can affect the final trial outputs. Nevertheless, despite the many challenges and uncertainties, it seems opportune and scientifically sound to further advance both scientific tools and regulatory frameworks for the development of these therapies, since the future products might offer unique therapeutic opportunities and equip the medical community with additional means for combating major human diseases.

## 6. Conclusions and Future Perspectives

AD is a complex, multifactorial disease. Although not a life-threatening condition, AD has a severe impact on the patient’s quality of life and is often associated with numerous medical and mental health comorbidities. Our understanding of AD and its pathophysiology has made major advances in the last decade, with detailed insights on the complex interplay between epidermal barrier dysfunction and immune system activation. Moreover, technological advances improved our ability to identify and characterize skin microbial communities, enhancing our knowledge on the disrupted host–microbiota relationship in AD. Recent reports provided evidence for introducing skin microbiota dysbiosis as one of the key features of the disease initiation and progression, paving the way for the development of novel therapeutic interventions. Probiotic and prebiotic preparations, as well as skin microbiota transplantation, are finding their way to clinical applications with promising results in AD management. However, more studies are needed to evaluate the influence of systemically and locally applied therapies to skin microbiota as well as to assess the mechanisms through which the effects are achieved. Additionally, the major challenge will be to translate these research findings into innovative new therapies and to overcome both scientific and regulatory challenges in developing microorganism-based medicinal products with an intended use in patients with AD.

## Figures and Tables

**Table 1:** Studies using probiotics and prebiotics in management of AD.

Study, Year	Study Type	Bacterial Strain	Administration	Animal Model	Outcome Summary
Kim et al., 2020 [93]	mouse model	*L. paracasei* KBL382	oral	NC/Nga mice DFE- and DNCB-induced AD	modulation of the immune response and gut microbiota
Kwon et al., 2018 [94]	mouse model	*L. sakei* WIKIM30	oral	BALB/c mice DNCB-induced AD	stimulation of Treg cell generation and suppression of T_H_2 inflammatory response, restoring the balance of gut microbiota
Lee et al., 2016 [95]	mouse model	*L. rhamnosus* IDCC 3201 tyndallizate (RHT3201)	oral	NC/Nga mice DFE-induced AD	less severe AD symptoms in comparison to controls, dose-dependent reductions in dermatitis scores
Kim et al., 2019 [96]	rat/mouse models	*L. plantarum* LM1004	oral	Sprague-Dawley rats, ddY mice histamine-induced AD DNFB-induced AD	inhibition of T_H_2 cell responses and activation of Treg immunoregulatory functions, increase of relative abundance of butyrate-generating microorganisms in the gut
**Study, Year**	**Study Type**	**Bacterial Strain**	**Administration**	**Participants (Age)**	**Outcome Summary**
Rosenfeldt et al., 2003 [97]	human	lyophilized *L. rhamnosus* 19070-2 and *L. reuteri* DSM 122460	oral	children (1–13 y)	moderate improvement in the clinical severity of eczema
Wickens et al., 2012 [98]	human	*L. rhamnosus*	oral	infants	reduced eczema prevalence
Wickens et al., 2013 [99]	human	*L. rhamnosus*	oral	children (<6 y)	significantly reduced cumulative eczema prevalence, decrease in SCORAD values and atopic sensitization
Wu et al., 2015 [100]	human	*L. rhamnosus*	oral	children (4–48 mos.)	decrease of SCORAD values and disease intensity
Han et al., 2012 [101]	human	*L. plantarum* CJLP133	oral	children (12 mos.–13 y)	decrease of SCORAD values, IFN-γ and IL-4
Weston et al., 2005 [102]	human	*L. fermentum* VRI-033 PCC	oral	children (6–18 mos.)	change in AD severity compared to placebo-treated individuals
Niccoli et al., 2014 [103]	human	*L. salivarious* LS01	oral	children	decrease of SCORAD values and significant improvement in itching intensity, both therapy benefits persisting after suspension of treatment
Matsumoto et al., 2014 [104]	human	*B. animalis* subsp. *lactis* LKM512	oral	adults	alleviated itch in AD patients and considerably improved the quality-of-life scores
Navarro-Lopez et al., 2018 [105]	human	*B. lactis* CECT 8145, *B. longum* CECT 7347, and *L. casei* CECT 9104	oral	children (4–17 y)	decrease of SCORAD values in patients with moderate AD
Lise et al., 2018 [106]	human	*B. lactis* HN019, *L. acidophilus* NCFM, *L. rhamnosus* HN001 and *L. paracasei* LPC-37	oral	children	evident response in treating severe AD with significant change in AD severity scores
Kim et al., 2014 [107]	human	*Lactobacillus* and *Bifidobacterium* species	oral	children and adults (1 mo.–65 y)	decrease of SCORAD values
Nakatsuji et al., 2017 [108]	human	topical application of commensal skin bacteria	topical	adults	protective effect against pathogen species (reduced *S. aureus* colonization due to selective AMPs secreted by commensal CoNS), improvement of clinical symptoms and decreased inflammation
Nakatsuji et al., 2021 [80]	human	*S. hominis* A9 (ShA9)	topical	adults	fewer adverse events associated with AD, inhibited expression of mRNA for psmα
Myles et al., 2018 [109]	human	*R. mucosa*	topical	children and adults	significant decrease in SCORAD and pruritus, reduction in disease severity and no adverse effects or complications
Blanchet-Rethoré et al., 2017 [110]	human	heat-treated *L. johnsonii*	topical	adults	clinical improvement of AD symptoms in patients with moderate AD
Di Marzio et al., 2003 [111]	human	sonicated *S. thermophilus*	topical	adults	significant improvement in skin barrier integrity, erythema, scaling and pruritus
Gueniche et al., 2008 [112]	human	lysate of *V. filiformis*	topical	children and adults	clinical improvement in patients with AD, decreased SCORAD values and pruritus
Chang et al., 2016 [113]	human	multiple strains of bacteria	topical	children (>1 y)	decrease of SCORAD values
Passeron et al., 2006 [114]	human	*L. rhamnosus* Lcr35 plus prebiotics	topical	children (>2 y)	improved AD symptoms and decreased SCORAD values
Aldaghi et al., 2020 [115]	human	*L. rhamnosus*, *L. euteri* and *B. infantis* or vitamin D3	topical	infants	significantly decreased SCORAD values

## References

[1] Scharschmidt T.C., Fischbach M.A. (2013). What Lives On Our Skin: Ecology, Genomics and Therapeutic Opportunities Of the Skin Microbiome. Drug Discov. Today Dis. Mech..

[2] SanMiguel A., Grice E.A. (2015). Interactions between host factors and the skin microbiome. Cell Mol. Life Sci..

[3] Grice E.A., Kong H.H., Conlan S., Deming C.B., Davis J., Young A.C., Program N.C.S., Bouffard G.G., Blakesley R.W., Murray P.R. (2009). Topographical and temporal diversity of the human skin microbiome. Science.

[4] Grice E.A., Segre J.A. (2011). The skin microbiome. Nat. Rev. Microbiol..

[5] Costello E.K., Lauber C.L., Hamady M., Fierer N., Gordon J.I., Knight R. (2009). Bacterial community variation in human body habitats across space and time. Science.

[6] Timm C.M., Loomis K., Stone W., Mehoke T., Brensinger B., Pellicore M., Staniczenko P.P.A., Charles C., Nayak S., Karig D.K. (2020). Isolation and characterization of diverse microbial representatives from the human skin microbiome. Microbiome.

[7] Oh J., Byrd A.L., Deming C., Conlan S., Program N.C.S., Kong H.H., Segre J.A. (2014). Biogeography and individuality shape function in the human skin metagenome. Nature.

[8] Oh J., Byrd A.L., Park M., Program N.C.S., Kong H.H., Segre J.A. (2016). Temporal Stability of the Human Skin Microbiome. Cell.

[9] Findley K., Oh J., Yang J., Conlan S., Deming C., Meyer J.A., Schoenfeld D., Nomicos E., Park M. (2013). Topographic diversity of fungal and bacterial communities in human skin. Nature.

[10] Prohic A., Sadikovic T.J., Krupalija-Fazlic M., Kuskunovic-Vlahovljak S. (2016). Malassezia species in healthy skin and in dermatological conditions. Int. J. Dermatol..

[11] Abdillah A., Khelaifia S., Raoult D., Bittar F., Ranque S. (2020). Comparison of Three Skin Sampling Methods and Two Media for Culturing Malassezia Yeast. J. Fungi.

[12] Zhang E., Tanaka T., Tajima M., Tsuboi R., Nishikawa A., Sugita T. (2011). Characterization of the skin fungal microbiota in patients with atopic dermatitis and in healthy subjects. Microbiol. Immunol..

[13] Rafat Z., Hashemi S.J., Ahamdikia K., Daie Ghazvini R., Bazvandi F. (2017). Study of skin and nail Candida species as a normal flora based on age groups in healthy persons in Tehran-Iran. J. Mycol. Med..

[14] Foulongne V., Sauvage V., Hebert C., Dereure O., Cheval J., Gouilh M.A., Pariente K., Segondy M., Burguiere A., Manuguerra J.C. (2012). Human skin microbiota: High diversity of DNA viruses identified on the human skin by high throughput sequencing. PLoS ONE.

[15] Hannigan G.D., Meisel J.S., Tyldsley A.S., Zheng Q., Hodkinson B.P., SanMiguel A.J., Minot S., Bushman F.D., Grice E.A. (2015). The human skin double-stranded DNA virome: Topographical and temporal diversity, genetic enrichment, and dynamic associations with the host microbiome. mBio.

[16] Shi B., Bangayan N.J., Curd E., Taylor P.A., Gallo R.L., Leung D.Y.M., Li H. (2016). The skin microbiome is different in pediatric versus adult atopic dermatitis. J. Allergy Clin. Immunol..

[17] Jo J.H., Deming C., Kennedy E.A., Conlan S., Polley E.C., Ng W.I., Program N.C.S., Segre J.A., Kong H.H. (2016). Diverse Human Skin Fungal Communities in Children Converge in Adulthood. J. Investig. Dermatol..

[18] Byrd A.L., Belkaid Y., Segre J.A. (2018). The human skin microbiome. Nat. Rev. Microbiol..

[19] Jurakic Toncic R., Jakasa I., Sun Y., Hurault G., Ljubojevic Hadzavdic S., Tanaka R.J., Pavicic B., Balic A., Zuzul K., Petkovic M. (2021). Stratum corneum markers of innate and T helper cell-related immunity and their relation to the disease severity in Croatian patients with atopic dermatitis. J. Eur. Acad. Dermatol. Venereol..

[20] Hulpusch C., Tremmel K., Hammel G., Bhattacharyya M., de Tomassi A., Nussbaumer T., Neumann A.U., Reiger M., Traidl-Hoffmann C. (2020). Skin pH-dependent Staphylococcus aureus abundance as predictor for increasing atopic dermatitis severity. Allergy.

[21] Rehbinder E.M., Advocaat Endre K.M., Lodrup Carlsen K.C., Asarnoj A., Stensby Bains K.E., Berents T.L., Carlsen K.H., Gudmundsdottir H.K., Haugen G., Hedlin G. (2020). Predicting Skin Barrier Dysfunction and Atopic Dermatitis in Early Infancy. J. Allergy Clin. Immunol. Pract..

[22] Tsakok T., Marrs T., Mohsin M., Baron S., du Toit G., Till S., Flohr C. (2016). Does atopic dermatitis cause food allergy? A systematic review. J. Allergy Clin. Immunol..

[23] Gustafsson D., Sjöberg O., Foucard T. (2000). Development of allergies and asthma in infants and young children with atopic dermatitis--a prospective follow-up to 7 years of age. Allergy.

[24] Spergel J.M., Paller A.S. (2003). Atopic dermatitis and the atopic march. J. Allergy Clin. Immunol..

[25] Zheng T., Yu J., Oh M.H., Zhu Z. (2011). The atopic march: Progression from atopic dermatitis to allergic rhinitis and asthma. Allergy Asthma Immunol. Res..

[26] Schneider L., Hanifin J., Boguniewicz M., Eichenfield L.F., Spergel J.M., Dakovic R., Paller A.S. (2016). Study of the Atopic March: Development of Atopic Comorbidities. Pediatr. Dermatol..

[27] Bieber T., D’Erme A.M., Akdis C.A., Traidl-Hoffmann C., Lauener R., Schappi G., Schmid-Grendelmeier P. (2017). Clinical phenotypes and endophenotypes of atopic dermatitis: Where are we, and where should we go?. J. Allergy Clin. Immunol..

[28] Williamson S., Merritt J., De Benedetto A. (2020). Atopic dermatitis in the elderly: A review of clinical and pathophysiological hallmarks. Br. J. Dermatol..

[29] Tanei R. (2020). Atopic Dermatitis in Older Adults: A Review of Treatment Options. Drugs Aging.

[30] Hanifin J.M., Rajka G. (1980). Diagnostic Features of Atopic-Dermatitis. Acta Derm. Venereol..

[31] Andersen R.M., Thyssen J.P., Maibach H.I. (2016). Qualitative vs. quantitative atopic dermatitis criteria-in historical and present perspectives. J. Eur. Acad. Dermatol. Venereol..

[32] Williams H.C., Burney P.G., Hay R.J., Archer C.B., Shipley M.J., Hunter J.J., Bingham E.A., Finlay A.Y., Pembroke A.C., Graham-Brown R.A. (1994). The U.K. Working Party’s Diagnostic Criteria for Atopic Dermatitis. I. Derivation of a minimum set of discriminators for atopic dermatitis. Br. J. Dermatol..

[33] Leshem Y.A., Hajar T., Hanifin J.M., Simpson E.L. (2015). What the Eczema Area and Severity Index score tells us about the severity of atopic dermatitis: An interpretability study. Br. J. Dermatol..

[34] Chopra R., Vakharia P.P., Sacotte R., Patel N., Immaneni S., White T., Kantor R., Hsu D.Y., Silverberg J.I. (2017). Relationship between EASI and SCORAD severity assessments for atopic dermatitis. J. Allergy Clin. Immunol..

[35] Silverberg J.I., Lei D., Yousaf M., Janmohamed S.R., Vakharia P.P., Chopra R., Chavda R., Gabriel S., Patel K.R., Singam V. (2021). What are the best endpoints for Eczema Area and Severity Index and Scoring Atopic Dermatitis in clinical practice? A prospective observational study. Br. J. Dermatol..

[36] Torres T., Ferreira E.O., Goncalo M., Mendes-Bastos P., Selores M., Filipe P. (2019). Update on Atopic Dermatitis. Acta Med. Port.

[37] Kantor R., Silverberg J.I. (2017). Environmental risk factors and their role in the management of atopic dermatitis. Expert Rev. Clin. Immunol..

[38] Weidinger S., Beck L.A., Bieber T., Kabashima K., Irvine A.D. (2018). Atopic dermatitis. Nat. Rev. Dis. Primers.

[39] Nedoszytko B., Reszka E., Gutowska-Owsiak D., Trzeciak M., Lange M., Jarczak J., Niedoszytko M., Jablonska E., Romantowski J., Strapagiel D. (2020). Genetic and Epigenetic Aspects of Atopic Dermatitis. Int. J. Mol. Sci..

[40] Weidinger S., Illig T., Baurecht H., Irvine A.D., Rodriguez E., Diaz-Lacava A., Klopp N., Wagenpfeil S., Zhao Y., Liao H. (2006). Loss-of-function variations within the filaggrin gene predispose for atopic dermatitis with allergic sensitizations. J. Allergy Clin. Immunol..

[41] Kawasaki H., Nagao K., Kubo A., Hata T., Shimizu A., Mizuno H., Yamada T., Amagai M. (2012). Altered stratum corneum barrier and enhanced percutaneous immune responses in filaggrin-null mice. J. Allergy Clin. Immunol..

[42] van Smeden J., Bouwstra J.A. (2016). Stratum Corneum Lipids: Their Role for the Skin Barrier Function in Healthy Subjects and Atopic Dermatitis Patients. Curr. Probl. Dermatol..

[43] Salimi M., Barlow J.L., Saunders S.P., Xue L., Gutowska-Owsiak D., Wang X., Huang L.C., Johnson D., Scanlon S.T., McKenzie A.N. (2013). A role for IL-25 and IL-33-driven type-2 innate lymphoid cells in atopic dermatitis. J. Exp. Med..

[44] Tsakok T., Woolf R., Smith C.H., Weidinger S., Flohr C. (2019). Atopic dermatitis: The skin barrier and beyond. Br. J. Dermatol..

[45] Malik K., Heitmiller K.D., Czarnowicki T. (2017). An Update on the Pathophysiology of Atopic Dermatitis. Dermatol. Clin..

[46] Mashiko S., Mehta H., Bissonnette R., Sarfati M. (2017). Increased frequencies of basophils, type 2 innate lymphoid cells and Th2 cells in skin of patients with atopic dermatitis but not psoriasis. J. Dermatol. Sci..

[47] Su C., Yang T., Wu Z., Zhong J., Huang Y., Huang T., Zheng E. (2017). Differentiation of T-helper cells in distinct phases of atopic dermatitis involves Th1/Th2 and Th17/Treg. Eur. J. Inflamm..

[48] Novak N., Bieber T., Hoffmann M., Folster-Holst R., Homey B., Werfel T., Sager A., Zuberbier T. (2012). Efficacy and safety of subcutaneous allergen-specific immunotherapy with depigmented polymerized mite extract in atopic dermatitis. J. Allergy Clin. Immunol..

[49] Wilson S.R., The L., Batia L.M., Beattie K., Katibah G.E., McClain S.P., Pellegrino M., Estandian D.M., Bautista D.M. (2013). The epithelial cell-derived atopic dermatitis cytokine TSLP activates neurons to induce itch. Cell.

[50] Oetjen L.K., Mack M.R., Feng J., Whelan T.M., Niu H., Guo C.J., Chen S., Trier A.M., Xu A.Z., Tripathi S.V. (2017). Sensory Neurons Co-opt Classical Immune Signaling Pathways to Mediate Chronic Itch. Cell.

[51] Kong H.H., Oh J., Deming C., Conlan S., Grice E.A., Beatson M.A., Nomicos E., Polley E.C., Komarow H.D., Program N.C.S. (2012). Temporal shifts in the skin microbiome associated with disease flares and treatment in children with atopic dermatitis. Genome Res..

[52] Geoghegan J.A., Irvine A.D., Foster T.J. (2018). Staphylococcus aureus and Atopic Dermatitis: A Complex and Evolving Relationship. Trends Microbiol..

[53] Williams M.R., Nakatsuji T., Gallo R.L. (2017). Staphylococcus aureus: Master Manipulator of the Skin. Cell Host Microbe.

[54] Fyhrquist N., Muirhead G., Prast-Nielsen S., Jeanmougin M., Olah P., Skoog T., Jules-Clement G., Feld M., Barrientos-Somarribas M., Sinkko H. (2019). Microbe-host interplay in atopic dermatitis and psoriasis. Nat. Commun..

[55] Totte J.E., van der Feltz W.T., Hennekam M., van Belkum A., van Zuuren E.J., Pasmans S.G. (2016). Prevalence and odds of Staphylococcus aureus carriage in atopic dermatitis: A systematic review and meta-analysis. Br. J. Dermatol..

[56] Di Domenico E.G., Cavallo I., Bordignon V., Prignano G., Sperduti I., Gurtner A., Trento E., Toma L., Pimpinelli F., Capitanio B. (2018). Inflammatory cytokines and biofilm production sustain Staphylococcus aureus outgrowth and persistence: A pivotal interplay in the pathogenesis of Atopic Dermatitis. Sci. Rep..

[57] Fleury O.M., McAleer M.A., Feuillie C., Formosa-Dague C., Sansevere E., Bennett D.E., Towell A.M., McLean W.H.I., Kezic S., Robinson D.A. (2017). Clumping Factor B Promotes Adherence of Staphylococcus aureus to Corneocytes in Atopic Dermatitis. Infect. Immun..

[58] Ong P.Y., Ohtake T., Brandt C., Strickland I., Boguniewicz M., Ganz T., Gallo R.L., Leung D.Y.M. (2002). Endogenous antimicrobial peptides and skin infections in atopic dermatitis. N. Engl. J. Med..

[59] Hata T.R., Kotol P., Boguniewicz M., Taylor P., Paik A., Jackson M., Nguyen M., Kabigting F., Miller J., Gerber M. (2010). History of eczema herpeticum is associated with the inability to induce human beta-defensin (HBD)-2, HBD-3 and cathelicidin in the skin of patients with atopic dermatitis. Br. J. Dermatol..

[60] Feuillie C., Vitry P., McAleer M.A., Kezic S., Irvine A.D., Geoghegan J.A., Dufrene Y.F. (2018). Adhesion of Staphylococcus aureus to Corneocytes from Atopic Dermatitis Patients Is Controlled by Natural Moisturizing Factor Levels. mBio.

[61] Nakatsuji T., Chen T.H., Two A.M., Chun K.A., Narala S., Geha R.S., Hata T.R., Gallo R.L. (2016). Staphylococcus aureus Exploits Epidermal Barrier Defects in Atopic Dermatitis to Trigger Cytokine Expression. J. Investig. Dermatol..

[62] Williams M.R., Nakatsuji T., Sanford J.A., Vrbanac A.F., Gallo R.L. (2017). Staphylococcus aureus Induces Increased Serine Protease Activity in Keratinocytes. J. Investig. Dermatol..

[63] Hon K.L., Tsang Y.C., Pong N.H., Leung T.F., Ip M. (2016). Exploring Staphylococcus epidermidis in atopic eczema: Friend or foe?. Clin. Exp. Dermatol..

[64] Fitz-Gibbon S., Tomida S., Chiu B.H., Nguyen L., Du C., Liu M., Elashoff D., Erfe M.C., Loncaric A., Kim J. (2013). Propionibacterium acnes strain populations in the human skin microbiome associated with acne. J. Investig. Dermatol..

[65] Lo C.W., Lai Y.K., Liu Y.T., Gallo R.L., Huang C.M. (2011). Staphylococcus aureus hijacks a skin commensal to intensify its virulence: Immunization targeting beta-hemolysin and CAMP factor. J. Invest. Dermatol..

[66] Wollenberg M.S., Claesen J., Escapa I.F., Aldridge K.L., Fischbach M.A., Lemon K.P. (2014). Propionibacterium-produced coproporphyrin III induces Staphylococcus aureus aggregation and biofilm formation. mBio.

[67] Kim J.E., Kim H.S. (2019). Microbiome of the Skin and Gut in Atopic Dermatitis (AD): Understanding the Pathophysiology and Finding Novel Management Strategies. J. Clin. Med..

[68] Nowicka D., Nawrot U. (2019). Contribution of *Malassezia* spp. to the development of atopic dermatitis. Mycoses.

[69] Scharschmidt T.C., Vasquez K.S., Truong H.A., Gearty S.V., Pauli M.L., Nosbaum A., Gratz I.K., Otto M., Moon J.J., Liese J. (2015). A Wave of Regulatory T Cells into Neonatal Skin Mediates Tolerance to Commensal Microbes. Immunity.

[70] Kennedy E.A., Connolly J., Hourihane J.O., Fallon P.G., McLean W.H.I., Murray D., Jo J.H., Segre J.A., Kong H.H., Irvine A.D. (2017). Skin microbiome before development of atopic dermatitis: Early colonization with commensal staphylococci at 2 months is associated with a lower risk of atopic dermatitis at 1 year. J. Allergy Clin. Immunol..

[71] Iwase T., Uehara Y., Shinji H., Tajima A., Seo H., Takada K., Agata T., Mizunoe Y. (2010). Staphylococcus epidermidis Esp inhibits Staphylococcus aureus biofilm formation and nasal colonization. Nature.

[72] Sugimoto S., Iwamoto T., Takada K., Okuda K., Tajima A., Iwase T., Mizunoe Y. (2013). Staphylococcus epidermidis Esp degrades specific proteins associated with Staphylococcus aureus biofilm formation and host-pathogen interaction. J. Bacteriol..

[73] Zipperer A., Konnerth M.C., Laux C., Berscheid A., Janek D., Weidenmaier C., Burian M., Schilling N.A., Slavetinsky C., Marschal M. (2016). Human commensals producing a novel antibiotic impair pathogen colonization. Nature.

[74] Nakatsuji T., Hata T.R., Tong Y., Cheng J.Y., Shafiq F., Butcher A.M., Salem S.S., Brinton S.L., Rudman Spergel A.K., Johnson K. (2021). Development of a human skin commensal microbe for bacteriotherapy of atopic dermatitis and use in a phase 1 randomized clinical trial. Nat. Med..

[75] Ramsey M.M., Freire M.O., Gabrilska R.A., Rumbaugh K.P., Lemon K.P. (2016). Staphylococcus aureus Shifts toward Commensalism in Response to Corynebacterium Species. Front. Microbiol..

[76] Shu M., Wang Y., Yu J., Kuo S., Coda A., Jiang Y., Gallo R.L., Huang C.M. (2013). Fermentation of Propionibacterium acnes, a commensal bacterium in the human skin microbiome, as skin probiotics against methicillin-resistant Staphylococcus aureus. PLoS ONE.

[77] Li H., Goh B.N., Teh W.K., Jiang Z., Goh J.P.Z., Goh A., Wu G., Hoon S.S., Raida M., Camattari A. (2018). Skin Commensal Malassezia globosa Secreted Protease Attenuates Staphylococcus aureus Biofilm Formation. J. Investig. Dermatol..

[78] Wollenberg A., Barbarot S., Bieber T., Christen-Zaech S., Deleuran M., Fink-Wagner A., Gieler U., Girolomoni G., Lau S., Muraro A. (2018). Consensus-based European guidelines for treatment of atopic eczema (atopic dermatitis) in adults and children: Part I. J. Eur. Acad. Dermatol. Venereol..

[79] Kim B.E., Leung D.Y.M. (2018). Significance of Skin Barrier Dysfunction in Atopic Dermatitis. Allergy Asthma Immunol. Res..

[80] Goh S.W., Jamil A., Safian N., Md Nor N., Muhammad N., Saharudin N.L. (2020). A randomized half-body, double blind, controlled trial on the effects of a pH-modified moisturizer vs. standard moisturizer in mild to moderate atopic dermatitis. An. Bras. Dermatol..

[81] Flores G.E., Seite S., Henley J.B., Martin R., Zelenkova H., Aguilar L., Fierer N. (2014). Microbiome of Affected and Unaffected Skin of Patients With Atopic Dermatitis Before and After Emollient Treatment. J. Drugs Dermatol..

[82] Drucker A.M., Eyerich K., de Bruin-Weller M.S., Thyssen J.P., Spuls P.I., Irvine A.D., Girolomoni G., Dhar S., Flohr C., Murrell D.F. (2018). Use of systemic corticosteroids for atopic dermatitis: International Eczema Council consensus statement. Br. J. Dermatol..

[83] Norris D.A. (2005). Mechanisms of action of topical therapies and the rationale for combination therapy. J. Am. Acad. Dermatol..

[84] Luger T., Paller A.S., Irvine A.D., Sidbury R., Eichenfield L.F., Werfel T., Bieber T. (2021). Topical therapy of atopic dermatitis with a focus on pimecrolimus. J. Eur. Acad. Dermatol. Venereol..

[85] Wongpiyabovorn J., Soonthornchai W., Wilantho A., Palasuk M., Payungporn S., Sodsai P., Poomipak W., Weschawalit S., Ruchusatsawat K., Baillie G.S. (2019). Effect of tacrolimus on skin microbiome in atopic dermatitis. Allergy.

[86] Bessa G.R., Quinto V.P., Machado D.C., Lipnharski C., Weber M.B., Bonamigo R.R., D’Azevedo P.A. (2016). Staphylococcus aureus resistance to topical antimicrobials in atopic dermatitis. An. Bras. Dermatol..

[87] Kwon S., Choi J.Y., Shin J.W., Huh C.H., Park K.C., Du M.H., Yoon S., Na J.I. (2019). Changes in Lesional and Non-lesional Skin Microbiome During Treatment of Atopic Dermatitis. Acta Derm. Venereol..

[88] Wollenberg A., Barbarot S., Bieber T., Christen-Zaech S., Deleuran M., Fink-Wagner A., Gieler U., Girolomoni G., Lau S., Muraro A. (2018). Consensus-based European guidelines for treatment of atopic eczema (atopic dermatitis) in adults and children: Part II. J. Eur. Acad. Dermatol. Venereol..

[89] Kim W.K., Jang Y.J., Han D.H., Jeon K., Lee C., Han H.S., Ko G. (2020). Lactobacillus paracasei KBL382 administration attenuates atopic dermatitis by modulating immune response and gut microbiota. Gut Microbes.

[90] Kwon M.S., Lim S.K., Jang J.Y., Lee J., Park H.K., Kim N., Yun M., Shin M.Y., Jo H.E., Oh Y.J. (2018). Lactobacillus sakei WIKIM30 Ameliorates Atopic Dermatitis-Like Skin Lesions by Inducing Regulatory T Cells and Altering Gut Microbiota Structure in Mice. Front. Immunol..

[91] Lee S.H., Yoon J.M., Kim Y.H., Jeong D.G., Park S., Kang D.J. (2016). Therapeutic effect of tyndallized Lactobacillus rhamnosus IDCC 3201 on atopic dermatitis mediated by down-regulation of immunoglobulin E in NC/Nga mice. Microbiol. Immunol..

[92] Kim I.S., Lee S.H., Kwon Y.M., Adhikari B., Kim J.A., Yu D.Y., Kim G.I., Lim J.M., Kim S.H., Lee S.S. (2019). Oral Administration of beta-Glucan and Lactobacillus plantarum Alleviates Atopic Dermatitis-Like Symptoms. J. Microbiol. Biotechnol..

[93] Rosenfeldt V., Benfeldt E., Nielsen S.D., Michaelsen K.F., Jeppesen D.L., Valerius N.H., Paerregaard A. (2003). Effect of probiotic Lactobacillus strains in children with atopic dermatitis. J. Allergy Clin. Immunol..

[94] Wickens K., Black P., Stanley T.V., Mitchell E., Barthow C., Fitzharris P., Purdie G., Crane J. (2012). A protective effect of Lactobacillus rhamnosus HN001 against eczema in the first 2 years of life persists to age 4 years. Clin. Exp. Allergy.

[95] Wickens K., Stanley T.V., Mitchell E.A., Barthow C., Fitzharris P., Purdie G., Siebers R., Black P.N., Crane J. (2013). Early supplementation with Lactobacillus rhamnosus HN001 reduces eczema prevalence to 6 years: Does it also reduce atopic sensitization?. Clin. Exp. Allergy.

[96] Wu Y.J., Wu W.F., Hung C.W., Ku M.S., Liao P.F., Sun H.L., Lu K.H., Sheu J.N., Lue K.H. (2017). Evaluation of efficacy and safety of Lactobacillus rhamnosus in children aged 4-48 months with atopic dermatitis: An 8-week, double-blind, randomized, placebo-controlled study. J. Microbiol. Immunol. Infect..

[97] Han Y., Kim B., Ban J., Lee J., Kim B.J., Choi B.S., Hwang S., Ahn K., Kim J. (2012). A randomized trial of Lactobacillus plantarum CJLP133 for the treatment of atopic dermatitis. Pediatr. Allergy Immunol..

[98] Weston S., Halbert A., Richmond P., Prescott S.L. (2005). Effects of probiotics on atopic dermatitis: A randomised controlled trial. Arch. Dis. Child..

[99] Niccoli A.A., Artesi A.L., Candio F., Ceccarelli S., Cozzali R., Ferraro L., Fiumana D., Mencacci M., Morlupo M., Pazzelli P. (2014). Preliminary Results on Clinical Effects of Probiotic Lactobacillus salivarius LS01 in Children Affected by Atopic Dermatitis. J. Clin. Gastroenterol..

[100] Matsumoto M., Ebata T., Hirooka J., Hosoya R., Inoue N., Itami S., Tsuji K., Yaginuma T., Muramatsu K., Nakamura A. (2014). Antipruritic effects of the probiotic strain LKM512 in adults with atopic dermatitis. Ann. Allergy Asthma Immunol..

[101] Navarro-Lopez V., Ramirez-Bosca A., Ramon-Vidal D., Ruzafa-Costas B., Genoves-Martinez S., Chenoll-Cuadros E., Carrion-Gutierrez M., Horga de la Parte J., Prieto-Merino D., Codoner-Cortes F.M. (2018). Effect of Oral Administration of a Mixture of Probiotic Strains on SCORAD Index and Use of Topical Steroids in Young Patients With Moderate Atopic Dermatitis: A Randomized Clinical Trial. JAMA Dermatol..

[102] Lise M., Mayer I., Silveira M. (2018). Use of probiotics in atopic dermatitis. Rev. Assoc. Med. Bras..

[103] Kim S.O., Ah Y.M., Yu Y.M., Choi K.H., Shin W.G., Lee J.Y. (2014). Effects of probiotics for the treatment of atopic dermatitis: A meta-analysis of randomized controlled trials. Ann. Allergy Asthma Immunol..

[104] Nakatsuji T., Chen T.H., Narala S., Chun K.A., Two A.M., Yun T., Shafiq F., Kotol P.F., Bouslimani A., Melnik A.V. (2017). Antimicrobials from human skin commensal bacteria protect against Staphylococcus aureus and are deficient in atopic dermatitis. Sci. Transl. Med..

[105] Myles I.A., Earland N.J., Anderson E.D., Moore I.N., Kieh M.D., Williams K.W., Saleem A., Fontecilla N.M., Welch P.A., Darnell D.A. (2018). First-in-human topical microbiome transplantation with Roseomonas mucosa for atopic dermatitis. JCI Insight.

[106] Blanchet-Rethore S., Bourdes V., Mercenier A., Haddar C.H., Verhoeven P.O., Andres P. (2017). Effect of a lotion containing the heat-treated probiotic strain Lactobacillus johnsonii NCC 533 on Staphylococcus aureus colonization in atopic dermatitis. Clin. Cosmet Investig. Dermatol..

[107] Di Marzio L., Centi C., Cinque B., Masci S., Giuliani M., Arcieri A., Zicari L., De Simone C., Cifone M.G. (2003). Effect of the lactic acid bacterium Streptococcus thermophilus on stratum corneum ceramide levels and signs and symptoms of atopic dermatitis patients. Exp. Dermatol..

[108] Gueniche A., Knaudt B., Schuck E., Volz T., Bastien P., Martin R., Rocken M., Breton L., Biedermann T. (2008). Effects of nonpathogenic gram-negative bacterium Vitreoscilla filiformis lysate on atopic dermatitis: A prospective, randomized, double-blind, placebo-controlled clinical study. Br. J. Dermatol..

[109] Chang Y.S., Trivedi M.K., Jha A., Lin Y.F., Dimaano L., Garcia-Romero M.T. (2016). Synbiotics for Prevention and Treatment of Atopic Dermatitis: A Meta-analysis of Randomized Clinical Trials. JAMA Pediatr..

[110] Passeron T., Lacour J.P., Fontas E., Ortonne J.P. (2006). Prebiotics and synbiotics: Two promising approaches for the treatment of atopic dermatitis in children above 2 years. Allergy.

[111] Aldaghi M., Tehrani H., Karrabi M., Abadi F.S., Sahebkar M. (2020). The effect of multistrain synbiotic and vitamin D3 supplements on the severity of atopic dermatitis among infants under 1 year of age: A double-blind, randomized clinical trial study. J. Dermatol. Treat..

[112] Pharmabiotic Research Institute. https://www.pharmabiotic.org/#mmps.

[113] Cordaillat-Simmons M., Rouanet A., Pot B. (2020). Live biotherapeutic products: The importance of a defined regulatory framework. Exp. Mol. Med..

[114] Margulis L.S., Fester R. (1991). Symbiosis as a Source of Evolutionary Innovation: Speciation and Morphogenesis.

[115] Theis K.R., Dheilly N.M., Klassen J.L., Brucker R.M., Baines J.F., Bosch T.C., Cryan J.F., Gilbert S.F., Goodnight C.J., Lloyd E.A. (2016). Getting the Hologenome Concept Right: An Eco-Evolutionary Framework for Hosts and Their Microbiomes. mSystems.

